# Reproducible changes in the gut microbiome suggest a shift in microbial and host metabolism during spaceflight

**DOI:** 10.1186/s40168-019-0724-4

**Published:** 2019-08-09

**Authors:** Peng Jiang, Stefan J. Green, George E. Chlipala, Fred W. Turek, Martha Hotz Vitaterna

**Affiliations:** 1 Center for Sleep and Circadian Biology, Department of Neurobiology, Northwestern University, Evanston, IL USA; 2 Sequencing Core, Research Resources Center, University of Illinois at Chicago, Chicago, IL USA

**Keywords:** Space environment, Microgravity, Cosmic radiation, 16S rRNA amplicon sequencing, RNA-seq

## Abstract

**Background:**

Space environment imposes a range of challenges to mammalian physiology and the gut microbiota, and interactions between the two are thought to be important in mammalian health in space. While previous findings have demonstrated a change in the gut microbial community structure during spaceflight, specific environmental factors that alter the gut microbiome and the functional relevance of the microbiome changes during spaceflight remain elusive.

**Methods:**

We profiled the microbiome using 16S rRNA gene amplicon sequencing in fecal samples collected from mice after a 37-day spaceflight onboard the International Space Station. We developed an analytical tool, named STARMAPs (Similarity Test for Accordant and Reproducible Microbiome Abundance Patterns), to compare microbiome changes reported here to other relevant datasets. We also integrated the gut microbiome data with the publically available transcriptomic data in the liver of the same animals for a systems-level analysis.

**Results:**

We report an elevated microbiome alpha diversity and an altered microbial community structure that were associated with spaceflight environment. Using STARMAPs, we found the observed microbiome changes shared similarity with data reported in mice flown in a previous space shuttle mission, suggesting reproducibility of the effects of spaceflight on the gut microbiome. However, such changes were not comparable with those induced by space-type radiation in Earth-based studies. We found spaceflight led to significantly altered taxon abundance in one order, one family, five genera, and six species of microbes. This was accompanied by a change in the inferred microbial gene abundance that suggests an altered capacity in energy metabolism. Finally, we identified host genes whose expression in the liver were concordantly altered with the inferred gut microbial gene content, particularly highlighting a relationship between host genes involved in protein metabolism and microbial genes involved in putrescine degradation.

**Conclusions:**

These observations shed light on the specific environmental factors that contributed to a robust effect on the gut microbiome during spaceflight with important implications for mammalian metabolism. Our findings represent a key step toward a better understanding the role of the gut microbiome in mammalian health during spaceflight and provide a basis for future efforts to develop microbiota-based countermeasures that mitigate risks to crew health during long-term human space expeditions.

**Electronic supplementary material:**

The online version of this article (10.1186/s40168-019-0724-4) contains supplementary material, which is available to authorized users.

## Background

The gastrointestinal microbiota plays an important role in mammalian health by interacting with host immune, metabolic, and neuropsychiatric functions [1, 2]. The space environment imposes many challenges to mammalian physiology, including functions known to interact with the gut microbiota in a bidirectional fashion. Specific space environmental factors, such as microgravity and radiation, are thought to alter the gut microbiota, representing a risk to astronaut health, especially during long-term spaceflight missions [3]. We previously studied the gut microbiome of a twin astronaut and found alterations during his 1-year mission onboard the International Space Station (ISS), which were not observed in his twin brother on Earth during the same period of time [4]. Similarly, spaceflight-associated microbiome changes were observed in mice flown on a space shuttle mission (STS-135) for 13 days [5]. However, the specific space environmental factors that influence the gut microbiome and the impact of these changes on host functions remain unknown.

**Fig. 1:**
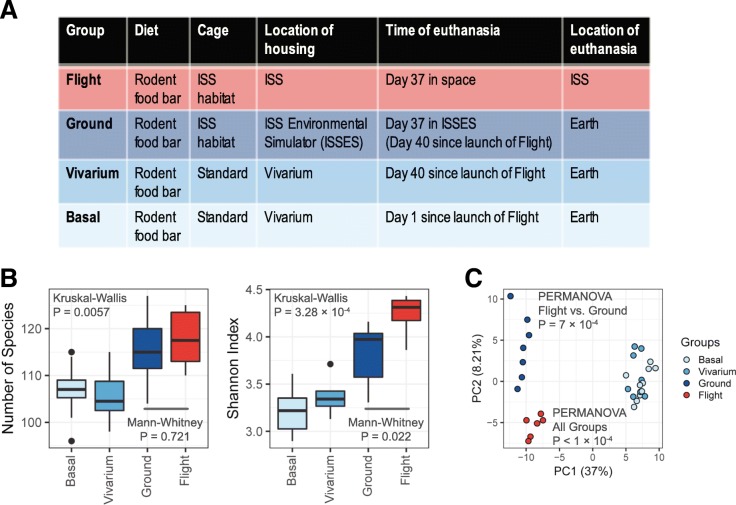
Microbial diversity of RR-1 fecal samples. **a** Animal groups involved in RR-1, highlighting group differences in the environmental conditions and durations (for details see the “Methods” section). The ISSES simulates the temperature, humidity, and CO_2_ partial pressure of the ISS environment based on data recorded onboard with a 3-day delay. **b** The number of microbial species observed in each sample (left) and the Shannon index (right) of microbial alpha diversity (i.e., within-sample diversity) varied among experimental groups of RR-1. **c** Analysis of beta diversity (i.e., between-sample diversity) using PCA on ILR-transformed relative abundance data found significant differences in the microbial composition among RR-1 experimental groups and specifically between Flight and Ground samples. Diversity analyses shown were performed using species-level data, and similar results were found at higher taxonomic levels as well (Additional file 1). Sample sizes in **b** and **c**: Basal, *n* = 10; Vivarium, *n* = 8; Ground, *n* = 7; Flight, *n* = 6

In 2014, NASA carried out the first ISS-based rodent research mission (RR-1), with the primary goal of validating hardware and operations for future rodent research missions [6]. RR-1 involved four groups of mice (Fig. 1a), and fecal samples from a subset of these animals were available, providing an opportunity to study the effects of spaceflight on the murine gut microbiome. Using 16S rRNA gene amplicon sequencing, we profiled the microbiome in these RR-1 samples and report spaceflight-associated changes in the gut microbial diversity and composition. We developed an analytical tool, Similarity Test for Accordant and Reproducible Microbiome Abundance Patterns (STARMAPs), to test the similarity of microbiome variations between two datasets. Using this method, we found the spaceflight-associated microbiome changes during RR-1 were similar to those during STS-135, suggesting a robust effect of spaceflight. However, when comparing the microbiome changes during RR-1 to those induced by space-type radiation in Earth-based studies [5, 7], we found no similarity, suggesting factors other than radiation are likely to drive the observed gut microbiome changes during spaceflight. By testing associations between inferred microbial gene content in the gut and the host liver transcriptome, we observed concordant variations suggesting potential interactions between the microbial metabolic capability and the host metabolism. Particularly, we highlight an association between the predicted abundance of bacterial genes involved in putrescine degradation in the gut and the expression of host genes involved in protein metabolism in the liver. These findings provide insights into the contributing factors of a reproducible change in the gut microbiota during spaceflight and the interactions between the gut microbiota and host metabolism in space.

## Results

### Spaceflight-associated changes in the gut microbial diversity and community structure

To evaluate the effect of spaceflight on the gut microbial alpha diversity (i.e., within-sample diversity), we computed the number of species observed in each sample (i.e., richness) and Shannon index (i.e., a diversity index accounting both evenness and richness) at the species level. We found that both the number of observed species and Shannon index significantly varied across RR-1 experimental groups (*P* = 0.0057 and *P* = 3.28 × 10^−4^, respectively, Kruskal-Wallis test) and were higher in Flight and Ground groups relative to Basal and Vivarium groups (Fig. 1b). Since the primary difference between Flight/Ground and Basal/Vivarium groups is the conditions of animal housing (i.e., habitat hardware, temperature, humidity, and CO_2_ levels; Fig. 1a), this observation indicates that the ISS rodent housing environment alters the richness and evenness of the murine gut microbial community. Interestingly, when comparing Flight and Ground animals, we found a slight increase in Shannon index (*P* = 0.022, Mann-Whitney test) but not in the number of observed species (*P* = 0.721, Mann-Whitney test) in Flight samples. Since the Ground animals were housed using the same ISS habitat equipment as the Flight animals under matched conditions of temperature, humidity, and CO_2_ levels in an ISS Environmental Simulator (ISSES), our observations suggest that factors specific to spaceflight induce an elevation in the evenness but not richness of the gut microbial community in mice.

We also observed significant differences in the gut microbial community structure among RR-1 groups (i.e., beta diversity analysis). Using principal component analysis (PCA) on isometric-log-ratio (ILR)-transformed species-level data, we found clear segregation of samples by the experimental group (*P* < 1 × 10^−4^, PERMANOVA; Fig. 1c). While the largest difference was between Flight/Ground samples and Basal/Vivarium samples, Flight samples were also significantly segregated from Ground samples (*P* = 7 × 10^−4^, PERMANOVA; Fig. 1c). Since the RR-1 groups were each associated with a distinct set of experimental conditions (Fig. 1a), we formulated the PERMANOVA test to replace animal groups with these associated factors in an additive model, in order to obtain an approximated estimation of the contributions of each condition to the overall variance of the gut microbial composition. Our analysis found that ISS housing conditions (i.e., habitat, temperature, humidity, and CO_2_ levels) accounted for 36.3% and spaceflight-specific factors accounted for 6.6% of the overall variance at the species level (for higher taxonomic levels, see Additional file 1). Thus, in addition to ISS housing conditions, we demonstrate that spaceflight-specific factors strongly modulate the composition of the gut microbiome.

### Reproducible effects of spaceflight on the murine gut microbiome composition

Spaceflight-associated changes in the gut microbiome composition have been reported in a recent study of fecal samples collected in mice onboard a space shuttle during the STS-135 mission in 2011 [5]. It is thus of interest to compare RR-1 data to STS-135 data, in order to test the reproducibility of spaceflight-associated changes in the gut microbiome. Comparing two different microbiome datasets in a formal statistical setting remains a challenge due to dataset-specific biases associated with biological and technical factors, such as animal or population cohorts, experimental conditions, sequencing strategies, data analysis methods, and many others. Despite these challenges, if changes in microbial compositions are reproducible, the microbial differential abundance patterns in two datasets are expected to involve a similar set of microbes with comparable amplitudes and directions of changes. We developed a statistical method, named STARMAPs, to capture this similarity by projecting samples from a second microbiome dataset onto the PCA axes that separate the groups of the first dataset. This method assumes that, when the group differences in two datasets are similar, the samples of each dataset in the microbial taxon space segregate by their respective groups in a similar fashion and that the PCA axes capturing the group segregation in the first dataset can also capture the similar group segregation in the second dataset.

To evaluate the performance of STARMAPs, we simulated pairs of datasets, each with 10% of the species that were differentially abundant with a given fold change (FC). The differential abundance patterns in a given pair of datasets were set to be either similar (i.e., involving the same set of differentially abundant species) or distinct (i.e., involving totally non-overlapping sets of differentially abundant species). We applied STARMAPs to each of the simulated pairs of datasets and compared the results to this “ground truth” for an evaluation of STARMAPs performance (Additional file 2: Figure S1). At the typical cutoff of omnibus *P* < 0.05, the specificity of STARMAPs was very high under all of the simulated conditions, while the sensitivity of the test varied in each of the scenarios. In the first simulation (Simulation 1; Additional file 2: Figure S1, left), we considered the influence of sample size in each of the dataset. Expectedly, when the differential abundance amplitude was small [i.e., log2(FC) = 1], the sensitivity of STARMAPs decreased as the sample size decreased. However, STARMAPs performed very well regardless of the sample size when the simulated amplitude of differential abundance was moderate or high [i.e., log2(FC) ≥ 2]. Since dataset-specific biological and technical biases are expected to cause differences in the amplitudes of differential abundance between datasets, in Simulation 2 (Additional file 2: Figure S1, middle), we introduced random variations into the differential abundance amplitude in the second dataset of the dataset pair, and tested whether STARMAPs can still capture the similarity between the pair of datasets. As the introduced variance increased, the sensitivity of STARMAPs decreased, particularly when the mean differential abundance was small [i.e., log2(FC) = 1]. However, when the mean differential abundance increased, the negative impact of this variation on test sensitivity reduced, indicating STARMAPs is well suited to identify differential abundance patterns that are similar but not necessarily identical in two datasets. Another expected consequence of dataset-specific biological and technical biases is the differences in the set of microbial species uncovered in each of the dataset, which was simulated in Simulation 3 (Additional file 2: Figure S1, right). As expected, the sensitivity of STARMAPs worsened as the proportion of commonly observed taxa in the pair of datasets decreased, due to loss of information. However, the decrease in sensitivity caused by low proportions of commonly observed taxa was in part compensated by the increase in the effect size. It is of interest to note that, when considering a similar effect in two microbiome datasets, it is likely that the proportion of taxa that are differentially abundant in both datasets is higher than the proportion of taxa that are commonly present in both datasets. In our simulation, all species in the second dataset have the same chance of not being found in the first dataset, and therefore, our simulation represented a harsher condition. Nevertheless, our simulations suggest that the performance of STARMAPs was satisfactory over a range of scenarios, particularly when the differential abundance amplitudes were relatively large.

**Fig. 2:**
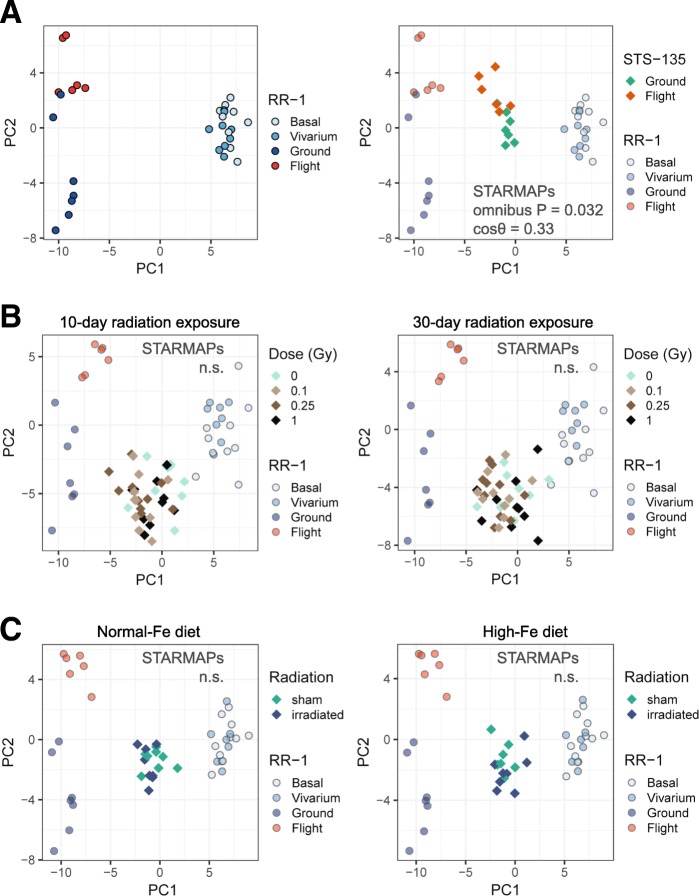
Comparison between microbiome variations during RR-1 to those during the STS-135 mission or induced by space-type radiation using STARMAPs. **a** Microbiome differences between RR-1 Flight and Ground samples were similar to microbiome differences between the flight and ground samples from the shuttle mission STS-135. A detailed description of STARMAPs is provided in the “Methods” section. Briefly, to compare group differences in the gut microbiome in one dataset to another, STARMAPs first performs PCA using samples from the first dataset alone (e.g., RR-1 data, left) and tests whether samples were segregated by the groups of interest (e.g., Flight vs. Ground) along the PCA axes. Then, samples from the second dataset (e.g., STS-135 data, RIGHT) are projected onto the same PCA axes and are tested for their group segregation along these PCA axes. As a third test, STARMAPs also evaluates the similarity in the directions of changes in the two datasets. It draws a line through the centers of the two groups of samples in each dataset to represent the group differences and tests whether the cosine of the angle, *θ*, between the lines in two datasets is significantly different from 0. When cos*θ* = 1, the microbiome changes in the two datasets are in exactly the same direction, and when cos*θ* = − 1, the microbiome changes in the two datasets are in exactly the opposite directions. Finally, STARMAPs uses an omnibus *P* value to summarize the three tests above to evaluate the overall similarity. Note that, while the graphs depict only PC1 and PC2, the tests were performed with all PCA axes. **b** Microbiome variations during RR-1 were compared to those induced by 10 days (left) or 30 days (right) of exposures to high-LET radiation exposure on Earth. **c** Microbiome variations in RR-1 mice were compared to those in rats exposed to low-LET radiation while fed on diets with either an adequate iron content (left) or a high-iron content (right). Note that STARMAPs uses random samplings from the Dirichlet distribution to estimate abundances of microbial taxa detected in one dataset but not the other. As a result, each time when comparing RR-1 data to another dataset, PCA of RR-1 samples gives very similar but not identical segregation patterns. Results shown here are at the species level, and similar results were observed at higher taxonomic levels as well (Additional file 1). Sample sizes of RR-1 data in **a**–**c**: Basal, *n* = 10; Vivarium, *n* = 8; Ground, *n* = 7; Flight, *n* = 6. Sample sizes of STS-135 data in **a**: Ground, *n* = 7; Flight, *n* = 6. Sample sizes of irradiated mice in **b**: *n* = 10 in each group. Sample sizes of irradiated rats in **c**: sham/Normal-Fe, *n* = 9; irradiated/Normal-Fe, *n* = 8; sham/High-Fe, *n* = 7; irradiated/High-Fe, *n* = 8

We next applied SATRMAPs to test whether spaceflight-associated changes in the gut microbiome during the RR-1 mission were similar to the STS-135 mission. Like RR-1, the mouse research onboard STS-135 involved a flight and a ground group with matched diet, habitat equipment, and environment (i.e., an environmental simulator was used), and a significant difference in the microbial community structure between the two groups was reported [5]. Using STARMAPs, we found that the differences in fecal microbial composition between the flight and the ground animals in the STS-135 mission were similar to those between RR-1 Flight and Ground animals (Fig. 2a; omnibus *P* = 0.032, STARMAPs). It can be noted that the directions of differences between Flight and Ground samples in the two missions were similar but not parallel with each other (cos*θ* = 0.33; *θ* is the angle between the directions of group differences in the two datasets). Aside from the technical differences in the microbiome profiling methods, this may be due to the differences in the mission duration (i.e., 13 days for STS-135 vs. 37 days for RR-1) or the sample collection strategies. STS-135 samples were collected from animals euthanized after return to Earth, while the RR-1 samples were collected from frozen carcasses of mice euthanized in orbit. Nonetheless, our findings indicate that space environmental factors produce robust and reproducible effects on the murine gut microbiome composition.

### Lack of similarity between spaceflight- and radiation-induced microbiome changes

We next sought to understand the contributions of specific space-associated factors to microbiome changes during spaceflight. It has been hypothesized that cosmic radiation is a unique environmental factor that can modulate the gut microbiome in space [3]. Previous Earth-based studies have indeed found changes in the gut microbiome in animals exposed to radiation that was similar in type to cosmic radiation. One study exposed mice to high-linear energy transfer (LET) radiation (600 MeV/n ^16^O) at doses of 0, 0.1, 0.25, or 1.0 Gy and reported changes in the gut microbiome composition and functional potential 10 and 30 days after the exposure [7]. Another study fed rats on either a high-iron diet or a diet with adequate iron for 14 days and then exposed the animals to low-LET radiation (^137^Cs fractionated radiation at 0.375 Gy/day) every other day for 16 days with a total dose of 3 Gy while continuing the assigned diets [5]. This study reported an altered relative abundance pattern of microbial orders that were associated with the diet and radiation exposure [5]. In order to test whether exposure to radiation significantly contributed to the microbiome changes during spaceflight, we used STARMAPs to compare the microbiome differences between RR-1 Flight and Ground groups to the space-type radiation-induced microbiome changes in these two Earth-based rodent studies (Fig. 2b, c). In both datasets, changes in the gut microbial community structure in response to radiation exposures were observed in our re-analysis at the species level (Additional file 3: Figure S2), confirming an effect of space-type radiation on the gut microbiome. However, the radiation-induced changes were not found to share significant similarity to those during RR-1 spaceflight (Fig. 2b, c). Although the exact nature of radiation exposure during RR-1 is unknown, radiation dosimetry data [8] recorded inside the space shuttle cabins during previous STS missions suggest that the total radiation dose and the dose rate (dose per day) during each mission were at least two and three magnitudes lower, respectively, than those used in the two Earth-based studies, which considered space environment beyond ISS and space shuttle orbits. It can be expected that the radiation exposure during RR-1 was likely to be similar to the STS missions, since ISS and space shuttles operate in similar obits. Therefore, our observation, together with the expected dose of RR-1 radiation exposure, suggests that space radiation alone during RR-1 is unlikely to be the predominant contributor to the observed microbiome changes and implies significant contributions from other space environmental factors.

### Spaceflight-associated changes in taxon abundance and inferred functional gene content

**Fig. 3:**
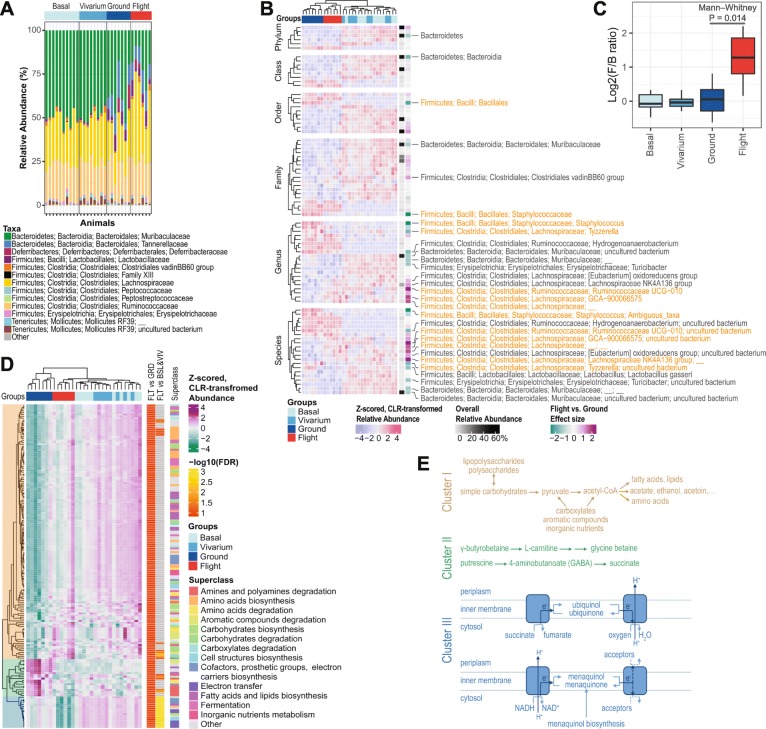
Differential abundance of microbial taxa and inferred gene content. **a** A stacked bar plot shows the relative abundance of microbial families uncovered in each sample, sorted by groups. For clarity, families with an overall abundance of less than 0.1% were summed into “Others”. **b** A heatmap depicts the differential abundance of microbial taxa that varied among RR-1 groups at FDR < 0.05. Rows (microbial taxa at each level) and columns (samples) were ordered by hierarchical clustering. The overall relative abundance of the taxa at a given taxonomic level is also noted as a sidebar of the heatmap. The effect sizes (i.e., the median ratio of between- and within-group differences) of differential taxon abundance comparing Flight samples to Ground samples were estimated using ALDEx2 and are plotted as a sidebar to highlight the differences between the two groups. Taxa that were significantly different (FDR < 0.05) between Flight and Ground samples are labeled in orange, and those suggestively different (*P* < 0.05 but FDR > 0.05) between Flight and Ground samples are labeled in dark grey. The identities of all the taxa in the heatmap and test statistics are provided in Additional file 1. **c** A boxplot showing the ratios between bacterial phyla *Firmicutes* and *Bacteroidetes* among RR-1 groups. **d** A heatmap showing inferred microbial metabolic pathways that were differentially abundant between Flight and Ground samples. Rows (microbial pathway) and columns (samples) were ordered by hierarchical clustering. Three clusters of microbial pathways were identified, and the dendrogram is color-shaded for each cluster. Microbial pathways are noted by their level-2 superclasses at the right side, and the identity of each pathway and test statistics are provided in Additional file 1. – log10(FDR) values are noted by the color scale on the right sidebars for the differential abundance test of each pathway between Flight and Ground samples (FLT vs. GRD) and between Flight samples and the combined Basal and Vivarium samples (FLT vs. BSL and VIV). **e** Simplified diagrams summarizing key microbial pathways of each cluster identified, colored accordingly as in **d**. Sample sizes in **a–d**: Basal, *n* = 10; Vivarium, *n* = 8; Ground, *n* = 7; Flight, *n* = 6

The altered microbial community structure among RR-1 groups was associated with altered relative abundance patterns that can be clearly seen at the family level (Fig. 3a). To identify specific microbial taxa affected by spaceflight, we used the ALDEx2 analysis package, which operates on centered-log-ratio (CLR) transformed sequencing data for a compositionally coherent inference of differential abundance [9]. Within the RR-1 dataset, at false discovery rate (FDR) < 0.05, 5 phyla, 6 classes, 10 orders, 15 families, 20 genera, and 18 species of bacteria were differentially abundant among the four experimental groups (Fig. 3b). Consistent with the PCA results, the predominant differences were observed between Flight/Ground samples and Basal/Vivarium samples, highlighting the strong impact of ISS rodent housing conditions on the gut microbiome composition. A number of taxa (1 order, 1 family, 5 genera, and 6 species), however, were significantly (FDR < 0.05, ALDEx2) differentially abundant between Flight and Ground groups, while an additional set of taxa (1 phylum, 1 class, 2 families, 6 genera, and 6 species) were suggestively (*P* < 0.05 but FDR > 0.05, ALDEx2) differentially abundant between the two groups (Fig. 3b). For example, the abundance of the bacteria in the phylum of *Bacteroidetes*, while was lower in the Ground/Flight animals compared to Basal/Vivarium animals, was also suggestively decreased (*P* < 0.05 but FDR > 0.05, ALDEx2) in the Flight animals compared to Ground animals. This change, together with a trend of elevated abundance of the *Firmicutes* phylum, led to a significantly increased *Firmicutes*-to-*Bacteroidetes* (F/B) ratio (Fig. 3c; *P* = 0.014, Mann-Whitney test, Flight vs. Ground), consistent with our previous findings in a twin astronaut during his 1-year spaceflight mission [4]. *Firmicutes* and *Bacteroidetes* are the two most common and abundant bacteria phyla found in the mammalian gastrointestinal tract. A change in the F/B ratio can be a sensitive marker, or serve as a proxy, of overall microbiome changes associated with many conditions. Examples include changes in the F/B ratio in patients with obesity [10], during aging in humans [11], and in response to dietary fiber particle size [12]. In addition, the relative abundance of *Tyzzerella* (a genus in the *Lachnospiraceae* family, *Clostridiales* order) was significantly decreased (FDR < 0.05, ALDEx2) in Flight animals compared to Ground animals, while the abundance of a few other genera of the *Lachnospiraceae* family were significantly (FDR < 0.05, ALDEx2) or suggestively (*P* < 0.05 but FDR > 0.05, ALDEx2) increased in Flight animals (Fig. 3b), revealing opposite effects of spaceflight on relatively close-related taxa. Similar patterns were observed in the *Ruminococcaceae* family, in which the *Ruminococcaceae UCG-010* genus showed a significantly increased (FDR < 0.05, ALDEx2) while the *Hydrogenoanaerobacterium* genus showed a suggestively decreased (*P* < 0.05 but FDR > 0.05, ALDEx2) abundance in the Flight animals compared to Ground animals. Finally, the relative abundance of the *Staphylococcus* genus of the *Bacillales* order was similar among Flight, Vivarium, and Basal samples, while the Ground samples appeared to be distinctively high (Fig. 3b), suggesting ISS rodent housing conditions and space-specific factors may induce opposite changes in the abundance of these microbes.

We next investigated the functional implication of these spaceflight-induced changes in gut microbial compositions. We used the software package PICRUSt2 to infer microbial gene content from 16S rRNA gene data and aggregated relative abundance of functional genes into metabolic pathways [13]. We then used ALDEx2 to identify differentially abundant pathways among RR-1 experimental groups. To capture the dominant functional features of spaceflight and ISS housing environment effects, we used a permissive threshold of FDR < 0.1. At this threshold, we found 619 pathways differentially abundant among groups (Additional file 1), 174 of which were differentially abundant between Flight and Ground animals (Fig. 3d). Hierarchical clustering of these 174 pathways based on the CLR-transformed relative abundance revealed three clusters, each with a unique differential abundance pattern and highlighting a specific mode of energy metabolism (Fig. 3d, e). Cluster I consists of a set of pathways that involve compounds used or produced by pyruvate fermentation, including carbohydrate degradation, aromatic compound degradation, carboxylate degradation, amino acid biosynthesis, lipid biosynthesis, and synthesis of polysaccharides. The relative abundance of genes in Cluster I pathways was low in Ground animals and higher in Flight animals. However, except for several pathways, Flight samples were not significantly different from the combined Basal and Vivarium samples (Fig. 3d and Additional file 1). This differential abundance pattern contrasted that of Cluster II, which contains a number of pathways related to utilizing amines as sources of nutrients and energy. The relative abundance of Cluster II pathway genes was high in Ground animals and was lower in Flight animals. In a few pathways (e.g., 4-aminobutanoate degradation I and III, urea degradation II, and putrescine degradation I; Fig. 3d and Additional file 1), gene abundance in Flight animals was also lower than Basal/Vivarium animals. Finally, Cluster III pathways are involved in electron transfer and biosynthesis of cofactors needed for aerobic and anaerobic respiration. Flight animals showed the lowest relative abundance of genes in this cluster, and Ground animals appeared to be intermediate between Flight and Basal/Vivarium animals. Taken together, our analysis of inferred microbial gene content revealed an increased abundance of fermentation genes and a decreased abundance of genes for respiration and amine degradation in Flight animals compared to the housing condition-matched Ground mice. This finding is consistent with a shift in energy metabolic capability in the gut microbiome during spaceflight.

It is worth noting that the choice of reference genome catalog influences the accuracy of microbiome gene content predictions. A recently developed integrated mouse gut metagenome catalog (iMGMC) has been shown to enhance the accuracy of PICRUSt predictions in mice [14], providing a useful resource for inferring the functional capability of the murine gut microbiome. We thus performed PICRUSt2 functional prediction with the iMGMC reference and compared the results with those obtained with the default reference, in order to ensure that the inference described above was robust. Using the iMGMC reference, PICRUSt2 analysis uncovered 592 of the 868 pathways that were uncovered with the default reference and 3 additional pathways (Additional file 4: Figure S3A; Additional file 1). This discrepancy is likely due to the fact that iMGMC reference, at its current stage, contains a small set of 16S rRNA-linked functional genomes (i.e., 484 genomes) that are specific to the murine gut microbiome, as opposed to the PICRUSt2 default reference, which contains a set of > 20,000 genomes of various origins. Despite this major difference, the predicted abundance of the commonly uncovered pathways and their differential abundance patterns between Flight and Ground animals derived using these two references were largely similar (Additional file 4: Figure S3B–D; Additional file 1). Given these observations, we continued our analysis with the functional predictions made using the PICRUSt2 default reference for a more inclusive analysis, in order to sufficiently capture the functional capability of the gut microbiome under the unique environment of spaceflight.

### Associations between the expression of host genes in the liver and inferred gene abundance of microbial metabolic pathways in the gut during spaceflight

**Fig. 4:**
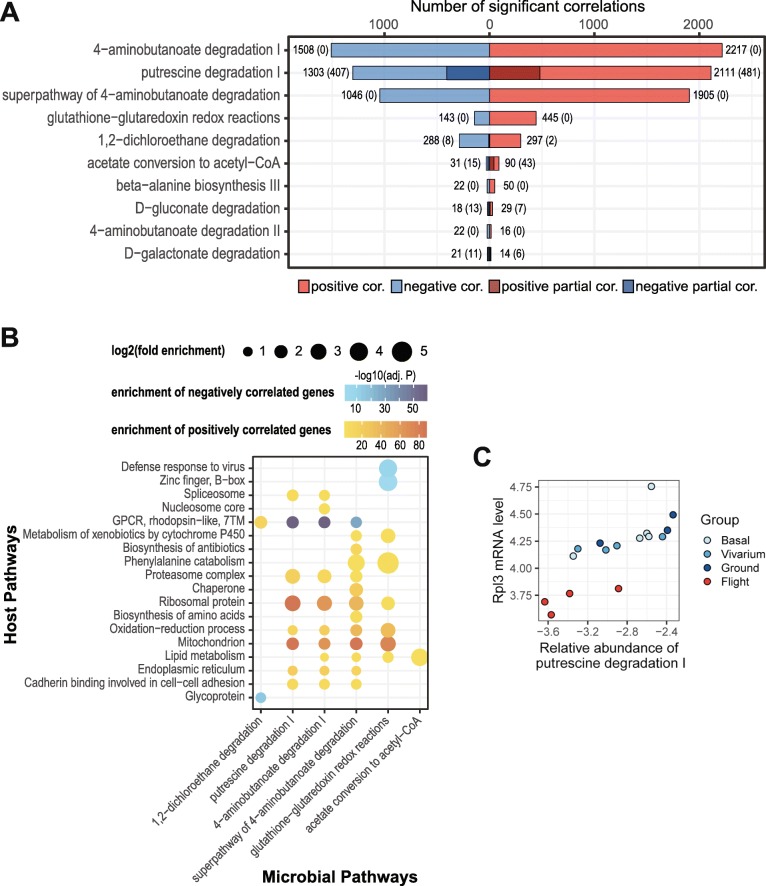
Relationships between inferred gene abundance of gut microbial pathways and gene expression in the host liver. **a** The number of host genes whose hepatic expression was significantly correlated (lighter color shades and numbers noted at end of the bars) and partially correlated (darker color shades and numbers noted in parenthesis) with the gene abundance of each of the microbial pathways. Top 10 pathways with the most numbers of correlated genes are shown. **b** Biological functions and pathways that were enriched with genes correlated with the gene abundance of each of the microbial pathways. **c** An example of correlations between the expression of a host gene in the liver and the inferred gene abundance of a microbial pathway in the gut. In this example, the overall correlation pattern between the expression of *Rpl3* (ribosomal protein L3) in the liver and the inferred gene abundance of microbial putrescine degradation I pathway were consistently observed within each experimental group, giving rise to a significant partial correlation. Only a subset of RR-1 animals has been profiled for both gut microbiome and liver transcriptome, and thus correlation analysis was performed with reduced sample sizes: Basal, *n* = 5; Vivarium, *n* = 4; Ground, *n* = 3; Flight, *n* = 4

To further understand the functional implications of spaceflight-associated changes in the gut microbiome, we utilized RNA-seq data in the liver of RR-1 mice stored in NASA’s GeneLab data repository [15, 16] and tested the correlations between the liver transcriptome of the host animal and the inferred relative gene abundance of microbial metabolic pathways in the gut, with the hypothesis that microbial metabolic potential and host metabolism are altered in coordination during spaceflight. We focused on the subset of microbial pathways that have been identified with differential inferred gene abundance between Flight and Ground animals (i.e., the 174 pathways in Fig. 3d), and performed the correlation analysis with multiple testing adjustment on a per-pathway basis in order to capture the dominant patterns of transcriptomic variations relevant to each microbial pathway of interest. The number of host genes significantly correlated (FDR < 0.1) with each microbial pathway were highly variable, ranging from a few thousand to only a few or even none (Fig. 4a and Additional file 1). For each microbial pathway with significantly correlated host genes, we identified biological processes and pathways that were enriched with those genes. This analysis revealed a number of host functions that covaried with gut microbial metabolism under the spaceflight and control conditions (Fig. 4b). Microbial 1,2-dichloroethane degradation (a Cluster I pathway in Fig. 3d) was positively correlated with genes encoding rhodopsin-like G-protein-coupled receptors (GPCRs) and was negatively correlated with genes encoding glycoproteins. In addition, microbial pathways of putrescine degradation, 4-aminobutanoate degradation, and glutathione-glutaredoxin redox reactions (Cluster II pathways in Fig. 3d) were positively correlated with host genes that were enriched in a number of pathways, most notably ribosome, proteasome, mitochondria, redox processes, lipid metabolism, and cell-cell adhesion. Lastly, microbial conversion of acetate to acetyl-CoA (a Cluster III pathway in Fig. 3d) was positively correlated with the expression of host genes involved in lipid metabolism, for which acetyl-CoA is a key intermediate.

We note that these correlations could be due to independent responses of the liver transcriptome and the gut microbiome to the ISS housing and spaceflight conditions, and are not necessarily indicative of interactions between liver functions and the gut microbial metabolic potential. Indeed, the majority of the correlations between microbial pathways and hepatic gene expression were no longer significant (FDR > 0.1) when partial correlations controlling for experimental groups were computed (Fig. 4a), suggesting these relationships reflected only a concurrence under the spaceflight and control conditions. Nonetheless, several potential microbial-host interactions were observed. The microbial pathway converting acetate to acetyl-CoA was associated with 121 genes, 48% of which remained significantly correlated (FDR < 0.1) when partial correlations were computed. In addition, about 26% of the genes correlated with the microbial putrescine degradation pathway remained significantly correlated (FDR < 0.1) after controlling for experimental groups. Enriched biological functions of these partially correlated genes confirmed a positive association between host protein metabolic genes (e.g., ribosome and proteasome; Fig. 4c and Additional file 1) in the liver and the capability of putrescine degradation by microbes in the gut. Putrescine is one of the most common polyamines that can be synthesized or uptaken by mammalian cells [17]. While polyamines are essential for many physiological functions, inhibited protein synthesis by excessive exogenous polyamines has been observed in a murine mammary carcinoma cell line [18]. Therefore, our observations raise an intriguing possibility that the decreased abundance of gut microbial putrescine degradation genes during spaceflight leads to a putrescine surplus, and in turn, to inhibition of host protein synthesis and metabolism.

## Discussion

Our analysis of the fecal samples from mice onboard the ISS and the three control groups on Earth during NASA’s RR-1 mission identified reproducible spaceflight-associated changes in the gut microbiome. These spaceflight-associated changes were linked to an altered transcriptome in the liver of the same animals. A few observations of the gut microbiome during spaceflight in the RR-1 mice reported here are consistent with our recent findings in a twin astronaut during his 1-year mission onboard the ISS, including an unchanged richness of microbial community, an altered community structure, and an elevated F/B ratio [4]. More importantly, using a newly developed statistical tool, STARMAPs, we tested the similarity in spaceflight-associated microbiome changes between RR-1 mice and mice onboard a previous shuttle mission STS-135 in a formal statistical setting, and we found that spaceflight-associated microbiome changes in the two missions were similar, despite the differences between the two missions in the durations of spaceflight, animal study protocol, as well as microbiome profiling and data processing methods. Together, our observations support a robust effect of spaceflight on the mammalian gut microbiome.

Importantly, the utilization of three diet-matched control groups in RR-1 allowed for attributing the observed microbiome variations to specific aspects of environmental factors associated with spaceflight missions. Particularly, Ground mice were housed using the same rodent habitat as Flight animals with matched temperature, humidity, and CO_2_ levels. While the Flight animals were traveling at high velocity and altitude when onboard the ISS, there were very few acceleration/deceleration events and the air pressure inside the ISS is kept at the sea level (i.e., same as where the control mice were housed). The major differences in the experimental conditions between Flight and Ground animals included the acceleration and vibration during launch as well as microgravity and cosmic radiation during spaceflight. The launch occurred 37 days prior to the time of sample collection and the duration was very short (i.e., minutes). Thus, the impact of launch on the gut microbiome, although cannot be excluded, is expected to be very small, and the observed microbiome changes between RR-1 Flight and Ground animals can be predominantly attributed to environmental conditions specific to spaceflight.

Although the effects of microgravity and cosmic radiation cannot be dissected experimentally using the current rodent research mission hardware, our study also presents a step toward an understanding of their contributions to the changes in the gut microbiome during spaceflight using statistical analysis with STARMAPs. Our analysis showed a lack of similarity between spaceflight-associated microbiome changes and those induced by exposures to space-type radiation on the ground. This lack of similarity implies that the gut microbiome is modulated largely by space environmental factors other than radiation during spaceflight. Given the differences in experimental conditions between Flight and Ground groups discussed above, we hypothesize that spaceflight-associated microbiome changes can be largely attributed to microgravity, which may influence microbial physiology and growth via low fluid shear dynamics [19, 20] and host physiological responses including altered digesta propulsion and digestive function [21], inflammation [22], fluid shift, and cardiovascular functions [23]. Microgravity also enables the animals to explore the three-dimensional space more easily in the rodent habitat, thus allowing behavioral changes that may be related to coping with stressors such as confinement. Future studies utilizing artificial gravity generated using a centrifuge onboard the ISS are needed to verify the effect of microgravity on the gut microbiome and its underlying mechanisms [24]. It is also worth noting that the lack of similarity between the effects of RR-1 spaceflight and space-type radiation may be due to the fact that the ISS operates in a low Earth orbit within the Van Allen Belts, and thus the animals were protected from the full impact of cosmic radiation. Indeed, radiation dosimetry data from previous space shuttle missions, which operated in the same or similar low Earth obits as the ISS, suggest the radiation exposure during RR-1 is expected to be magnitudes lower than those in the two datasets used in our analysis [5, 7, 8]. Nevertheless, given the observed effects of space-type radiation on the gut microbiome when the doses were much larger than expected for ISS and shuttle missions [5, 7], future studies are needed to understand how space radiation may alter the gut microbiome during space travels beyond Van Allen Belts.

In addition to spaceflight-associated changes, our study also observed pronounced differences in the gut microbiome composition between Basal/Vivarium and Flight/Ground groups, highlighting a strong effect of the rodent housing condition onboard the ISS. Many factors associated with this ISS housing condition may contribute to the observed differences. For example, the ISS rodent habitat, unlike the Basal/Vivarium cages, is equipped with wire-mesh grid surfaces and a special ventilation system to create continuous airflow to remove small particles (e.g., food, water, and animal waste) from the cage in order to keep the cage clean in microgravity. Grid surfaces are known to induce stress and changes in the gut microbiome composition [25]. In addition, the CO_2_ concentration is higher on ISS than on Earth, although the O_2_ level on ISS is kept at near sea-level values, and this condition was matched in Ground animals in the ISSES. Intermittent hypoxia has been shown to alter the gut microbiome compositions in mice [26, 27]. While it is unclear whether this ISS condition induces hypoxia, gene expression changes consistent with hypoxic responses have been reported in mice housed with ISS housing conditions compared to standard vivarium conditions [16, 28] and in a twin astronaut when onboard the ISS [4]. Another striking change in the gut microbiome associated with ISS housing conditions included an elevated alpha diversity in Flight/Ground animals compared to Basal/Vivarium animals. Although the gut microbiome in animals housed under a closed environment or under stress is generally expected to show a decreased alpha diversity [29], we speculate that the increased diversity in ISS habitat housed mice may be related to the constant airflow created by the ventilation system of the habitat, although other factors may also have contributed to this change.

Furthermore, it is worth noting that our study was limited by the availability of fecal samples only at the end of the mission, particularly given that animals of the same group were housed together in a single cage during the mission and a cage effect could confound our findings. However, the inclusion of Basal animals provided an assessment of gut microbiome prior to the mission. The lack of difference between Basal and Vivarium animals suggests that the gut microbiome was quite stable without exposures to ISS housing or spaceflight conditions and that the cage effect was likely to be minimal. Future rodent research missions designed to study the gut microbiome are expected to longitudinally monitor the gut microbiome before, during, and after spaceflight, in order to further characterize spaceflight-associated changes.

Finally, our study provided inference regarding the functional relevance of changes in the gut microbiome composition during spaceflight. We showed that spaceflight-associated changes in taxon abundance were accompanied by changes in the inferred gene abundance of microbial metabolic pathways, suggesting that an altered metabolic need may drive taxonomic changes in the gut microbiota under space environmental conditions. By analyzing transcriptomic data in the liver of the RR-1 animals, we identified host genes whose expression in the liver covaried with the inferred gene abundance of gut microbial metabolic pathways during spaceflight. Although this analysis only links the predicted microbial metabolic capability with transcriptional signatures implicating host metabolic state and does not establish interactions of metabolic activities between host and the gut microbiome, our observations suggested interesting candidates for future studies to mechanistically interrogate such interactions. In addition, most of the associations are likely due to the concurrence of independent responses of the host and microbiome to the spaceflight environment, as the associations were no longer significant after controlling the effects of experimental groups. However, we highlight here a spaceflight-associated decrease in the relative gene abundance of microbial putrescine degradation pathway, which was correlated with the expression of host genes involved in protein synthesis and degradation even after controlling for group effects, suggesting a potential host-microbial interaction that may contribute to a decline in protein metabolism in the host liver during spaceflight [30, 31]. Furthermore, some of the concurrent host and microbial responses during spaceflight, even though they may not directly interact with each other, could have important health implications. For example, the inferred abundance of genes encoding microbial glutathione-glutaredoxin redox enzymes was positively correlated with the hepatic expression of host genes involved in redox processes (Fig. 4b). These associations were not significant, however, when partial correlations were computed to control for effects of experimental groups (Fig. 4a). Thus, the data suggest that these particular host and microbial pathways were not likely interacting with each other. However, the decreased gene abundance of the microbial glutathione-glutaredoxin pathway and its covariation with the expression of the host redox-related genes during spaceflight suggest a decreased capability to maintain redox homeostasis in all cellular systems, exposing physiological functions to oxidative damage at a systems level [4, 32, 33].

## Conclusions

Taken together, our observations demonstrate a robust effect of the spaceflight on the gut microbiome, which may be attributed to specific space environmental factors, likely microgravity, and suggest an altered metabolic potential in the gut microbiota that was associated with the expression of metabolic genes in the host liver. We speculate that microgravity leads to an altered metabolic environment for the microbes in the gastrointestinal tract via mechanisms such as lowered fluid shear dynamics, altered digesta movement, as well as other physiological and behavioral responses of the host, and the gut microbiota adapts to such changes by shifting community structure and associated gene content, which may in turn influence host biological functions. As such, a change in the gut microbiota is a key component of mammalian adaptation to the space environment. Further characterizations and mechanistic studies of the complex interactions between the host and the gut microbiome during spaceflight are needed and may enable interventions allowing the microbial communities to adapt to the spaceflight-associated metabolic environment in the gut while avoiding harm or even conferring benefits to mammalian physiology. Such a strategy will mitigate risks to crew health and performance during future long-term spaceflight missions.

## Methods

### Fecal samples from RR-1 mice

Fecal samples from 32 RR-1 animals were obtained through NASA’s Biospecimen Sharing Program, and no live animals were involved in this study. The detailed information regarding the RR-1 mission is available through NASA’s Life Sciences Data Archive (https://lsda.jsc.nasa.gov/document/doc_detail/Doc13600) as well as previous publications [6]. Briefly, a single cohort of adult (16 weeks of age at the time of launch) female C57BL/6J mice were ordered from the Jackson Laboratory and housed under standard vivarium conditions before launch. All animals were fed on NASA’s spaceflight-approved rodent food bar starting at 24 days prior to the launch and throughout the entire study. Two weeks prior to the launch, animals were regrouped into cages of 10 animals, and a week later, one cage of 10 mice was selected as Flight animals and was launched on Sept. 21, 2014, with the SpaceX-4 resupply mission to the ISS. Mice arrived at the ISS 4 days later and were then transferred into the ISS rodent habitat and housed for a total of 37 days in space until euthanasia in orbit. Frozen carcasses (stored at − 80 °C) were returned to Earth and then were dissected for tissue collection, including the fecal materials dissected from the colon. While this sample collection protocol requires additional freeze/thaw cycles, which may influence microbiome profiling, it avoided the technically challenging detailed dissection in-orbit and is advantageous over sample collection from live-returned animals, which introduces confounding factors such as stress and condition changes associated with the return flight. In addition to Flight, three ground control groups (a cage of 10 mice per group) were selected from the same cohort. These include (1) a Basal group euthanized the day after the launch, (2) a Vivarium group kept in standard cages, and (3) a Ground group experienced a launch/transportation simulation and housed in the identical spaceflight habitat hardware placed in an ISSES chamber, which reproduced the temperature, CO2, and humidity environment of Flight animals based on 72-hour-delayed data collected on ISS. Ground animals were kept in ISSES for 37 days and were euthanized on day 40 since the launch of Flight animals. Vivarium animals were euthanized together with the Ground animals. Euthanasia, as well as tissue and fecal sample collection procedures (including the number of freeze/thaw cycles involved) for animals from the three control groups, were performed the same way as the Flight group. Fecal samples were available from a subset of the RR-1 mice, including 7 Flight, 7 Control, 8 Vivarium, and 10 Basal animals. Note that RR-1 also included another set of mice, known as the CASIS commercial mice, which were in space for 21–22 days [34]. Samples from these mice were not a part of NASA’s Biospecimen Sharing Program and were not included in our study.

### Microbiome characterization

Genomic DNA was extracted from individual fecal pellets using a Maxwell Tissue kit, implemented on a Maxwell 16 automated extraction robot (Promega, Madison, WI), as described previously [35]. The extracted DNA was PCR-amplified using primers (forward 515F GTGCCAGCMGCCGCGGTAA and reverse 926R CCGYCAATTYMTTTRAGTTT) targeting the V4–V5 variable regions of microbial 16S ribosomal RNA genes [36], using a two-stage targeted amplicon sequencing protocol [37]. The primers contained 5′ common sequence tags (known as common sequence 1 and 2, CS1 and CS2) [38] in addition to the 515F/926R sequences. First-stage PCR amplifications were performed in 10 μl reactions in 96-well plates, using the MyTaq HS 2X master mix. PCR conditions were 95 °C for 5 min, followed by 28 cycles of 95 °C for 30′′, 50 °C for 60′′, and 72 °C for 90′′. Subsequently, a second PCR amplification was performed in 10 μl reactions in 96-well plates. A master mix for the entire plate was made using the MyTaq HS 2X master mix. Each well received a separate primer pair with a unique 10-base barcode, obtained from the Access Array Barcode Library for Illumina (Fluidigm, South San Francisco, CA; Item# 100-4876). These AccessArray primers contained the CS1 and CS2 linkers at the 3′ ends of the oligonucleotides. Cycling conditions were as follows: 95 °C for 5 min, followed by 8 cycles of 95 °C for 30′′, 60 °C for 30′′, and 72 °C for 30′′. A final 7-min elongation step was performed at 72 °C. Samples were pooled in equal volume using an EpMotion5075 liquid handling robot (Eppendorf, Hamburg, Germany). The pooled libraries were purified using an AMPure XP cleanup protocol (0.6 ×, vol/vol; Agencourt, Beckmann-Coulter) to remove fragments smaller than 300 bp. The pooled libraries, with a 20% phiX spike-in, were loaded onto an Illumina MiniSeq mid-output flow cell (2 × 150 paired-end reads). Based on the distribution of reads per barcode, the amplicons (before purification) were re-pooled to generate a more balanced distribution of reads. The re-pooled libraries were again purified using the AMPure XP cleanup protocol to remove fragments smaller than 300 bp. The re-pooled libraries, with a 20% phiX spike-in, were loaded onto a MiSeq v3 flow cell and sequenced (2 × 300 paired-end reads; > 40,000 reads/sample requested) using an Illumina MiSeq sequencer. Fluidigm sequencing primers, targeting the CS1 and CS2 linker regions, were used to initiate sequencing. De-multiplexing of reads was performed on the instrument. Library preparation, pooling, size selection, and sequencing were performed at the University of Illinois at Chicago Sequencing Core (UICSQC).

Sequence data were processed through a QIIME (v1.8) pipeline. Briefly, forward and reverse reads were merged using PEAR [39]. Reads were then trimmed using a quality threshold of *P* = 0.01. Primer sequences were trimmed from the reads, and any reads lacking either primer were discarded. Reads with internal ambiguous nucleotides or less than 300 bp in length after trimming were also discarded. Reads were further filtered to exclude chimeric sequences identified using the USEARCH algorithm [40] as compared with the Greengenes_13_8 database [41]. The software package QIIME [42] was used to generate taxonomic summaries, employing a “sub-OTU” modification of the standard pipeline [43]. Briefly, all sequences were dereplicated to produce a list of unique sequences. All sequences that had an abundance of at least 10 counts were designated “seed” sequences, and USEARCH was used to find the nearest seed sequence for any non-seed sequence with a minimum identity threshold of 98%. The count threshold (i.e., 10 counts) for choosing seed sequences was set based on the distribution of the sequence data. When sequence data were binned based on the replicate number of a unique sequence and the total sequence count in each bin was plotted against the replicate number, we found that the inflection point on the curve falls just below a count of 10, below which the total sequence counts in the bins displayed a nearly exponential decay. Using this threshold, less than 2% of the unique sequences were used as seed sequences for the clustering, accounting for 58% of the sequence counts. Taxonomic annotations were assigned to each master sequence and independent low-abundance sequences using Silva_132 reference database, and sample-by-taxon abundance matrices at multiple taxonomic levels were generated for statistical analyses and data visualization. One Flight sample was excluded from all subsequent analysis due to an extremely low number of sequencing reads. The final dataset contains 6 Flight, 7 Control, 8 Vivarium, and 10 Basal samples (1 sample per animal).

### Diversity and differential abundance analysis

For alpha diversity, data was rarefied at 33.2 k reads per sample, and Shannon indexes were computed at each taxonomic level. Non-parametric statistical tests (i.e., Kruskal-Wallis and Mann-Whitney tests) were used to test for group differences. To perform a beta diversity analysis appropriate to the compositional nature of sequencing data [44, 45], we used PCA on ILR-transformed sequencing counts [46, 47]. The non-parametric PERMANOVA test [48], implemented in the adonis function of the R/vegan package (v2.5-2) was then used to identify group differences with 10,000 permutations.

To identify differentially abundant taxa, we applied ALDEx2 [9] (v1.12.0) at each taxonomic level. We focused on taxa with an overall relative abundance more than 0.01% and excluded low-abundance taxa from the differential abundance analysis. ALDEx2 performs CLR-transformation to the sequencing count data for a compositionally coherent inference and estimates *P* values and false discovery rates (FDR) from independent testing of Monte Carlo Dirichlet instances to control for type-I error due to the underestimated variance of low abundance taxa. Data at each taxonomic level was analyzed independently. While ALDEx2 provides both parametric and non-parametric test statistics, only non-parametric test results were reported in this study.

We inferred the microbial gene content from the taxa abundance using PICRUSt2 (https://github.com/picrust/picrust2; v2.0.0-b). PICRUSt2 is a significant expansion of PICRUSt [13] with a > 10 × larger reference genomes database and provides MetaCyc [49] pathway predictions comparable with typical shotgun metagenomics datasets. We used ALDEx2 to identify group differences in the inferred gene abundance of MetaCyc pathways. Differentially abundant taxa and inferred pathways were visualized in heatmaps and hierarchically clustered based on Euclidian distances of CLR-transformed data. To evaluate the influence of reference catalog on the prediction of microbial functional gene content, we replaced the default reference catalog in PICRUSt2 with a mouse gut microbiome specific reference catalog, the iMGMC reference [14]. The catalog files were downloaded from https://github.com/tillrobin/iMGMC/tree/master/PICRUSt. A phylogenetic tree was built from the downloaded 16S rRNA alignment file using RAxML-NG [50] (v0.8.0) with the GTR + G model and 50 bootstraps. The phylogenetic tree was provided together with all other iMGMC reference files to PICRUSt2. Since the functional genes predicted with iMGMC reference was in KEGG Orthology IDs, the gene IDs were converted to Enzyme Commission numbers using the R/KEGGREST package (v1.22.0) which provides a client interface to the KEGG REST server. For each KEGG Orthology IDs matched to multiple Enzyme Commission numbers, the predicted abundance was split equally to each Enzyme Commission number; for each Enzyme Commission number matched to multiple KEGG Orthology IDs, the summed abundance was used. After this conversion, predicted abundance for enzymes was provided to PICRUSt2 to aggregate into MetaCyc pathway abundance for a comparison with results obtained using the PICRUSt2 default reference.

### STARMAPs

We were interested in comparing microbiome changes in two different datasets, in order to test the reproducibility of space-induced changes in the microbiome as well as to associate the effects of candidate factors to the observed effects of spaceflight. Methods comparing the differential expression patterns in two transcriptomics datasets have been previously developed. These methods were based on testing the enrichment of a list of up/downregulated genes in one dataset against the pattern of genome-wide differential expression/abundance in another dataset, as exemplified by the method developed by the Connectivity Map project [51]. However, these methods do not perform well on microbiome datasets, especially those using 16S rRNA amplicon sequencing, due to the fact that a typical microbiome dataset uncovers only hundreds of taxa (as opposed to tens of thousands of genes in transcriptomics datasets) and a handful of differentially abundant taxa, leading to much reduced statistical power. To address this issue, we developed STARMAPs (Similarity Test for Accordant and Reproducible Microbiome Abundance Patterns), which does not depend on differential abundance or enrichment analyses but instead testing whether particular linear combinations of taxon abundance capture the group differences in two microbiome datasets in a similar fashion.

STARMAPs considers the taxon-by-sample tables from two microbiome datasets, ds1 and ds2. When the differential taxon abundance patterns in ds1 and ds2 are similar, the differentially abundant taxa in two datasets involve a similar set of taxa and the group differences of a given taxon in two datasets are comparable in magnitude and direction. Thus, it can be expected that, when ds1 and ds2 samples are plotted in the same microbial taxon space, the samples segregate by their respective groups in each dataset in a similar fashion. When applying a rotation of axes in PCA so that the first few PCs capture the group segregation in ds1, the similar group segregation in ds2 would also be apparent with the same PCs from the same axis rotation. To test this similarity in group segregation, STARMAPs first matches the microbial taxa at a given taxonomic level to include all taxa detected in either dataset, so that the samples of two datasets are in the same microbial taxon space. Taxa detected only in one dataset are filled with 0 counts in the other dataset. Next, a point estimate of relative abundance is obtained from the mean of 1000 Monte Carlo Dirichlet instances based on the counts with an added offset of 0.5. STARMAPs then ILR-transforms the data into the Euclidean space for both datasets and performs PCA using only ds1. The same rotation matrix from PCA of ds1 is applied to ds2 so that samples of ds2 are projected to the same PCA axes as ds1. Sample segregation patterns in the two datasets are then evaluated with this set of PCA axes. A significant similarity in the group differences is called by STARMAPs when the following three criteria are met simultaneously: (1) ds1 samples are segregated by the groups, evaluated by PERMANOVA of the first few PCs. (2) Group segregation in ds2 can be seen on the same PCA axes capturing the ds1 group segregation, also evaluated by PERMANOVA. In addition to the typical sample permutations used in PERMANOVA, a second permutation test, in which the taxon matching between ds1 and ds2 is randomized, is also used in order to ensure the specificity of the linear combination of microbial taxa in discriminating group differences. The larger *P* value from the two permutation tests is taken as the final *P* value. (3) The directions of changes in two datasets are not perpendicular to each other. To evaluate this, a line is drawn through the centers of the two groups being compared in each dataset, to represent the directions of change in the respective dataset. The cosine of the angle between the two lines (cos*θ*) is computed. Thus, when cos*θ* = 1, the directions of group differences in the two datasets are the same; when cos*θ* = − 1, the directions are the opposite of each other; and when cos*θ* = 0, the directions are perpendicular and the group differences in two datasets are not comparable. A bootstrap test is used to estimate Pr(cos*θ* = 0) as the test *P* value. Since calling similarity requires satisfaction of all three conditions described above, the rejection region of the overall hypothesis test is the intersection of the rejection regions of the component tests. Thus, the omnibus *P* value of this overall hypothesis test can be given using the Intersection-Union Test framework and computed as the supremum of the *P* values of the component tests [52]. We implemented STARMAPs (v2) in R (v3.5) and the script is available at GitHub (https://github.com/pjiang82/STARMAPs).

We tested the performance of STARMAPs using simulated datasets. Data simulations were performed as described by McMurdie and Holmes [53] using the fecal microbiome data from the “Global Patterns” dataset [54] for a realistic evaluation of STARMAPs performance. For each simulation, we considered two datasets, each with two groups and a sample size of *N* per group. We simulated four *N* samples at sequencing depths determined by the depths of randomly chosen samples in the Global Patterns dataset and randomly drew sequence counts to each of the microbial species according to the overall species abundance distribution of the fecal samples in the Global Patterns dataset. To simulate differential abundance patterns, we assumed that a moderate proportion (10%) of the microbial species were differentially abundant with a specified effect size ranging from small to large fold changes [log2(FC) = 1, 2, 3, or 4], and we applied the effect size to the randomly selected set of species. To simulate a pair of datasets with true similarity in the respective group difference, the effect size was applied to the same set of microbial species in each of the datasets. To simulate a pair of datasets with no similarity, the effect size was applied to distinct sets of species in two datasets, while keeping the number of species in each set the same. For each of the 12 evaluations shown in Additional file 2: Figure S1, 2000 pairs of datasets were simulated with an approximately 1:1 ratio for true similarity pairs and no similarity pairs. In Simulation 1, we tested the effects of sample size. We assumed the two datasets uncovered the exact same species (i.e., the proportion of species commonly found in two datasets is 1, or, overlap = 1) and the differential abundant species in two datasets changed with the same log2(FC) [i.e., no variation in log2(FC), or, *s* = 0]. In Simulation 2, we evaluated the effects of variable effect sizes between two datasets, while keeping the sample size *N* at 6 per group and the proportion of overlapping species between datasets at 1. While the same set of species in two datasets was set as differentially abundant, the log2FC applied to the first dataset was constant (i.e., 1, 2, 3, or 4) but the effect size applied to the second dataset is a normally distributed variable with a mean of 1, 2, 3, or 4 (same as in the first dataset) and a standard deviation *s* (*s* = 0.5, 1, 2, or 4). In Simulation 3, we evaluated effects of varying proportions of overlapping species found in two datasets, while setting the sample size *N* = 6 per group and the standard deviation of effect size applied to the second dataset *s* = 1. We simulated the differential abundance patterns in the same way as in Simulation 2, but randomly added a string of “xx” to the species names at a given proportion (1 - overlap) in the second dataset so that they cannot be matched with the species names in the first dataset. Codes used for the data simulations and performance evaluations are available at GitHub (https://github.com/pjiang82/STARMAPs).

We used STARMAPs to compare the microbiome differences between Flight and Ground animals during RR-1 to the spaceflight-associated differences during STS-135 and to radiation-induced changes in Earth-based studies. The raw 16S rRNA gene sequencing reads were downloaded from NCBI’s Sequence Read Archive (SRA) database. Sequencing data from STS-135 mice and rats exposed to low-LET radiation [5] were downloaded with the accession number SRP058196 but were processed separately. Sequencing data from mice exposed to high-LET radiation [7] were downloaded with the accession number SRP098151. Since the 16S rRNA gene sequencing was done with different primers and different settings, it is not possible to process the data in an identical manner as our RR-1 data. While using a closed-reference operational taxonomic unit (OTU) approach can reduce the impact of biases associated with different primer sets, it may also fail to capture the key variations of interest and is less intuitive given all other analyses were done with the typical open-reference OTU approach. In addition, our simulations have suggested that STARMAPs can tolerate some challenging conditions associated with dataset-specific biological and technical biases. Therefore, we processed these SRA datasets independently using the QIIME2 (https://qiime2.org/; v2018.2) pipeline. The SRP058196 dataset (i.e., STS-135 mice and low-LET irradiated rats) contains single-end sequencing data of the V1–V2 region of the bacterial 16S rRNA gene and was analyzed with the Deblur [55] plugin, which trimmed the sequences at a quality threshold of *P* < 1 × 10^−4^, removed chimeras and reads shorter than 200 bases, and assembled the sub-OTUs at 99% sequence identity. The SRP098151 dataset (i.e., high-LET irradiated mice) contains pair-end sequencing data of the V4 region of the 16S rRNA gene, and DADA2 [56] was used to denoise and dereplicate sequence reads with a quality filtering at *P* < 0.01 and chimera removal, before constructing the feature table at 99% sequence identity. For each dataset, taxonomic assignments were made according to Silva_132 reference database, and a sample-by-species abundance matrix was generated for analysis using STARMAPs in comparison with the RR-1 data. In this study, 10,000 permutations or bootstraps were used for each of the three composite tests in STARMAPs for all comparisons.

### Analysis of the liver transcriptome

The liver transcriptome has been profiled in a subset of RR-1 animals using RNA-seq, and the data is available via the NASA GeneLab database under the accession numbers GLDS-48 and GLDS-168. The GLDS-48 dataset does not include Basal and Vivarium animals and thus only contains half of the samples as in GLDS-168. We therefore only used the GLDS-168 dataset. The GLDS-168 dataset includes transcriptomics data from 20 RR-1 animals, 16 of which (including 5 Basal, 4 Vivarium, 3 Ground, and 4 Flight mice) were also studied for their fecal microbiome in this study. The purpose of the GLDS-168 dataset was to evaluate the utility of control RNA spike-ins in RNA-seq data analysis, and we only used data from sample aliquots without the added control RNA spike-ins. The detailed sample processing and sequencing procedures can be found in https://genelab-data.ndc.nasa.gov/genelab/accession/GLDS-168/. RNA sequencing reads were analyzed against the mouse reference transcriptome (GRCm38) using Salmon [57] (v0.10.2) to quantify transcript-level expression (measured as transcript per million, or TPM), which was then summarized into the gene level using tximport [58] (v1.8.0). We calculated Spearman’s correlations between gene-level expression (CLR-transformed TPM) and the PICRUSt2-inferred gene abundance of microbial pathways (also CLR-transformed), for each of the inferred microbial pathways that were differentially abundant between Flight and Ground group. FDRs were estimated using the Benjamini-Hochberg procedure, independently for correlations of each microbial pathway. This permissive approach was taken because our analysis was focused on a set of pre-selected microbial pathways and was interested to capture host gene expression that concordantly altered with each microbial pathway of interest. For each significant correlation between a host gene and a microbial pathway, partial correlations controlling for experimental groups were also computed. To compute partial correlations, we first fit the gene expression or microbial pathway gene abundance separately in robust linear models with the experimental groups. Spearman correlations were then computed using the residuals of these linear models. DAVID [59] (v6.8) was used to identify host functions enriched with genes that were correlated with each of the microbial pathways.

## Additional files

Additional file 1: The complete set of analysis results. (XLSX 1341 kb)

Additional file 2: **Figure S1.** Receiver operating characteristic (ROC) curves showing the performance of STARMAPs using simulated data. In each panel, STARMAPs was applied to 2000 simulated pairs of datasets that either shared a set of differentially abundant species (true positives) or involved distinct sets of differentially abundant species (true negatives), and the sensitivity (true positive fraction) and specificity (1 – false positive fraction) of STARMAPs were plotted at given STARMAPs omnibus P values ranging from 0 to 1. The enlarged point on each curve indicates the sensitivity and specificity estimated at omnibus P = 0.05. Simulations were done with varying amplitudes of differential abundance [i.e., effect size in log2(FC); rows] over a range of parameters representing different scenarios (columns). These varying parameters included the sample size (N) for each group, the variance (s) of the log2(FC) applied to the second dataset of a given pair of datasets, and the proportion of species commonly found in a given pair of datasets (overlap). (PDF 2000 kb)

Additional file 3: **Figure S2.** Radiation-induced changes in the community structure of gut microbiome in rodents. Beta diversity analyses of the two publically available datasets were performed using PCA on ILR-transformed relative abundance data at the species level. (**A**) Exposure to high-LET radiation (600 MeV/n ^16^O) at various doses altered the community structure of the gut microbiome in mice 10 or 30 days after the exposure (n = 10 in each group) [7]. Significant effects of time (P = 0.018, PERMANOVA) and dose (P < 0.0001, PERMANOVA) were observed. (**B**) Exposing rats to low-LET radiation (^137^Cs fractionated radiation at 0.375 Gy every other day, totaling at 3 Gy) induced a shift in the gut microbiome community structure (P = 0.0029, PERMANOVA) [5]. A diet-by-radiation interaction effect was also observed (P = 0.033, PERMANOVA). Sample sizes: sham/Normal-Fe, n = 9; irradiated/Normal-Fe, n = 8; sham/High-Fe, n = 7; irradiated/High-Fe, n = 8. (PDF 2280 kb)

Additional file 4: **Figure S3.** Comparison between inferred gene abundance of microbial pathways predicted with the PICRUSt default reference and iMGMC reference. (**A**) A Venn diagram showing the number of pathways uncovered with each of the reference catalogs. (**B**) Coefficients of pathway-wise correlations computed for each of the samples showing a high agreement in the predicted gene abundance of pathways uncovered by both reference catalogs. (**C**) Sample-wise correlations between the relative abundance predicted using the two reference catalogs were computed for each of the pathways, and a histogram is used here to show the distribution of correlation coefficients. CLR-transformed relative abundance was used to compute correlation coefficients. (**D**) A scatter plot showing the overall similarity (Pearson’s r = 0.61, P = 6.98 × 10^-61^) in the differential abundance effect sizes estimated by ALDEx2 using gene abundance predicted with either reference catalog. Each point denotes a pathway that commonly uncovered using both references, and points filled with colors indicate pathways found to be differentially abundant (FDR < 0.1, ALDEx2) when the abundance was predicted using either reference or both. The lower correlation coefficients in **C** and **D** compared to **B** was expected, as differences in the number of pathways uncovered with each reference influences the estimated total sequence abundance and thus the estimated relative abundance of each predicted pathway. (PDF 1300 kb)

## Data Availability

All data needed to evaluate the conclusions in the paper are present in the paper and the supplementary information. Raw 16S rRNA amplicon sequencing data of RR-1 samples have been deposited to NASA GeneLab database under the accession number GLDS-212 (https://genelab-data.ndc.nasa.gov/genelab/accession/GLDS-212). The sequencing data is also available through NCBI’s SRA database under the accession number SRP192647 (BioProject: PRJNA532760). The script of STARMAPs can be found at https://github.com/pjiang82/STARMAPs.

## Abbreviations

CLR: Centered-log-ratio
F/B: *Firmicutes*-to-*Bacteroidetes*
FC: Fold change
FDR: False discovery rate
GPCRs: G-protein-coupled receptors
ILR: Isometric-log-ratio
iMGMC: Integrated mouse gut metagenome catalog
ISS: International Space Station
ISSES: ISS Environment Simulator
LET: Linear energy transfer
NASA: National Aeronautics and Space Administration
OTU: Operational taxonomic unit
PCA: Principal component analysis
PERMANOVA: Permutational multivariate analysis of variance
RR-1: Rodent Research-1
STARMAPs: Similarity Test for Accordant and Reproducible Microbiome Abundance Patterns
TPM: Transcript per million
