# Supplementary material for: Reproducible changes in the gut microbiome suggest a shift in microbial and host metabolism during spaceflight
Source: Microbiome. 2019 Aug 9;7:113. doi: 10.1186/s40168-019-0724-4 (PMC6689164; doi:10.1186/s40168-019-0724-4)
Supplement: Supplementary file 2 — Figure S1. Receiver operating characteristic (ROC) curves showing the performance of STARMAPs using simulated data. In each panel, STARMAPs was applied to 2000 simulated pairs of datasets that either shared a set of differentially abundant species (true positives) or involved distinct sets of differentially abundant species (true negatives), and the sensitivity (true positive fraction) and specificity (1 – false positive fraction) of STARMAPs were plotted at given STARMAPs omnibus P values ranging from 0 to 1. The enlarged point on each curve indicates the sensitivity and specificity estimated at omnibus P = 0.05. Simulations were done with varying amplitudes of differential abundance [i.e., effect size in log2(FC); rows] over a range of parameters representing different scenarios (columns). These varying parameters included the sample size (N) for each group, the variance (s) of the log2(FC) applied to the second dataset of a given pair of datasets, and the proportion of species commonly found in a given pair of datasets (overlap). (PDF 2000 kb) [file 40168_2019_724_MOESM2_ESM.pdf]

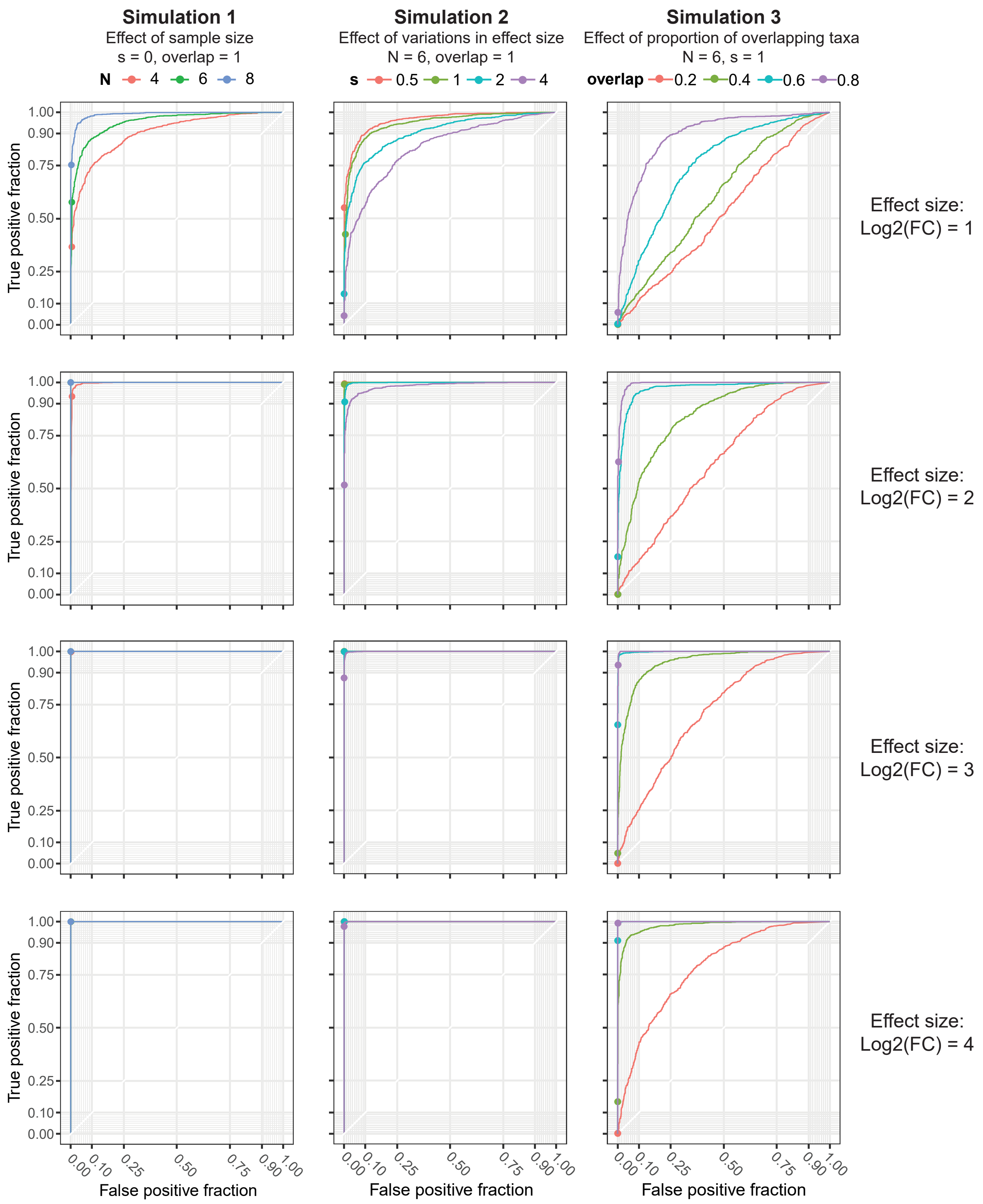

Figure S1. Receiver operating characteristic (ROC) curves showing the performance of STARMAPs using simulated data. In each panel, STARMAPs was applied to 2000 simulated pairs of datasets that either shared a set of differentially abundant species (true positives) or involved distinct sets of differentially abundant species (true negatives), and the sensitivity (true positive fraction) and specificity (1 – false positive fraction) of STARMAPs were plotted at given STARMAPs omnibus P values ranging from 0 to 1. The enlarged point on each curve indicates the sensitivity and specificity estimated at omnibus P = 0.05. Simulations were done with varying amplitudes of differential abundance [i.e., effect size in log2(FC); rows] over a range of parameters representing different scenarios (columns). These varying parameters included the sample size (N) for each group, the variance (s) of the log2(FC) applied to the second dataset of a given pair of datasets, and the proportion of species commonly found in a given pair of datasets (overlap).
