# Supplementary material for: Reproducible changes in the gut microbiome suggest a shift in microbial and host metabolism during spaceflight
Source: Microbiome. 2019 Aug 9;7:113. doi: 10.1186/s40168-019-0724-4 (PMC6689164; doi:10.1186/s40168-019-0724-4)
Supplement: Supplementary file 3 — Figure S2. Radiation-induced changes in the community structure of gut microbiome in rodents. Beta diversity analyses of the two publically available datasets were performed using PCA on ILR-transformed relative abundance data at the species level. (A) Exposure to high-LET radiation (600 MeV/n 16O) at various doses altered the community structure of the gut microbiome in mice 10 or 30 days after the exposure (n = 10 in each group) [7]. Significant effects of time (P = 0.018, PERMANOVA) and dose (P < 0.0001, PERMANOVA) were observed. (B) Exposing rats to low-LET radiation (137Cs fractionated radiation at 0.375 Gy every other day, totaling at 3 Gy) induced a shift in the gut microbiome community structure (P = 0.0029, PERMANOVA) [5]. A diet-by-radiation interaction effect was also observed (P = 0.033, PERMANOVA). Sample sizes: sham/Normal-Fe, n = 9; irradiated/Normal-Fe, n = 8; sham/High-Fe, n = 7; irradiated/High-Fe, n = 8. (PDF 2280 kb) [file 40168_2019_724_MOESM3_ESM.pdf]

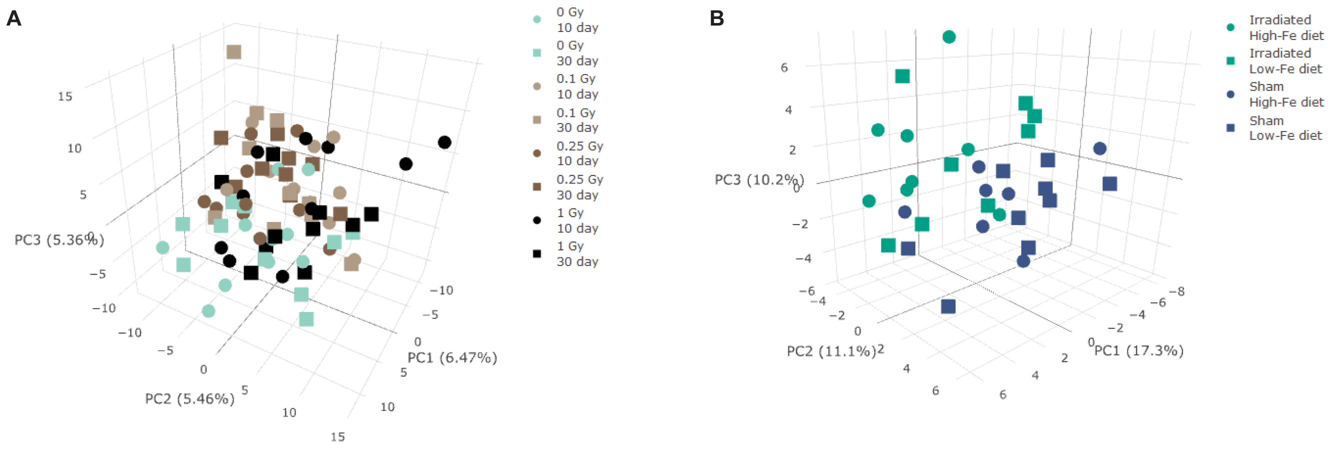

Figure S2. Radiation-induced changes in the community structure of gut microbiome in rodents. Beta diversity analyses of the two publically available datasets were performed using PCA on ILR-transformed relative abundance data at the species level. (**A**) Exposure to high-LET radiation (600 MeV/n ^{16}O) at various doses altered the community structure of the gut microbiome in mice 10 or 30 days after the exposure (n = 10 in each group) [7]. Significant effects of time (P = 0.018, PERMANOVA) and dose (P < 0.0001, PERMANOVA) were observed. (**B**) Exposing rats to low-LET radiation (^{137}Cs fractionated radiation at 0.375 Gy every other day, totaling at 3 Gy) induced a shift in the gut microbiome community structure (P = 0.0029, PERMANOVA) [5]. A diet-by-radiation interaction effect was also observed (P = 0.033, PERMANOVA). Sample sizes: sham/Normal-Fe, n = 9; irradiated/Normal-Fe, n = 8; sham/High-Fe, n = 7; irradiated/High-Fe, n = 8.
