# Supplementary material for: Reproducible changes in the gut microbiome suggest a shift in microbial and host metabolism during spaceflight
Source: Microbiome. 2019 Aug 9;7:113. doi: 10.1186/s40168-019-0724-4 (PMC6689164; doi:10.1186/s40168-019-0724-4)
Supplement: Supplementary file 4 — Figure S3. Comparison between inferred gene abundance of microbial pathways predicted with the PICRUSt default reference and iMGMC reference. (A) A Venn diagram showing the number of pathways uncovered with each of the reference catalogs. (B) Coefficients of pathway-wise correlations computed for each of the samples showing a high agreement in the predicted gene abundance of pathways uncovered by both reference catalogs. (C) Sample-wise correlations between the relative abundance predicted using the two reference catalogs were computed for each of the pathways, and a histogram is used here to show the distribution of correlation coefficients. CLR-transformed relative abundance was used to compute correlation coefficients. (D) A scatter plot showing the overall similarity (Pearson’s r = 0.61, P = 6.98 × 10^-61^) in the differential abundance effect sizes estimated by ALDEx2 using gene abundance predicted with either reference catalog. Each point denotes a pathway that commonly uncovered using both references, and points filled with colors indicate pathways found to be differentially abundant (FDR < 0.1, ALDEx2) when the abundance was predicted using either reference or both. The lower correlation coefficients in C and D compared to B was expected, as differences in the number of pathways uncovered with each reference influences the estimated total sequence abundance and thus the estimated relative abundance of each predicted pathway. (PDF 1300 kb) [file 40168_2019_724_MOESM4_ESM.pdf]

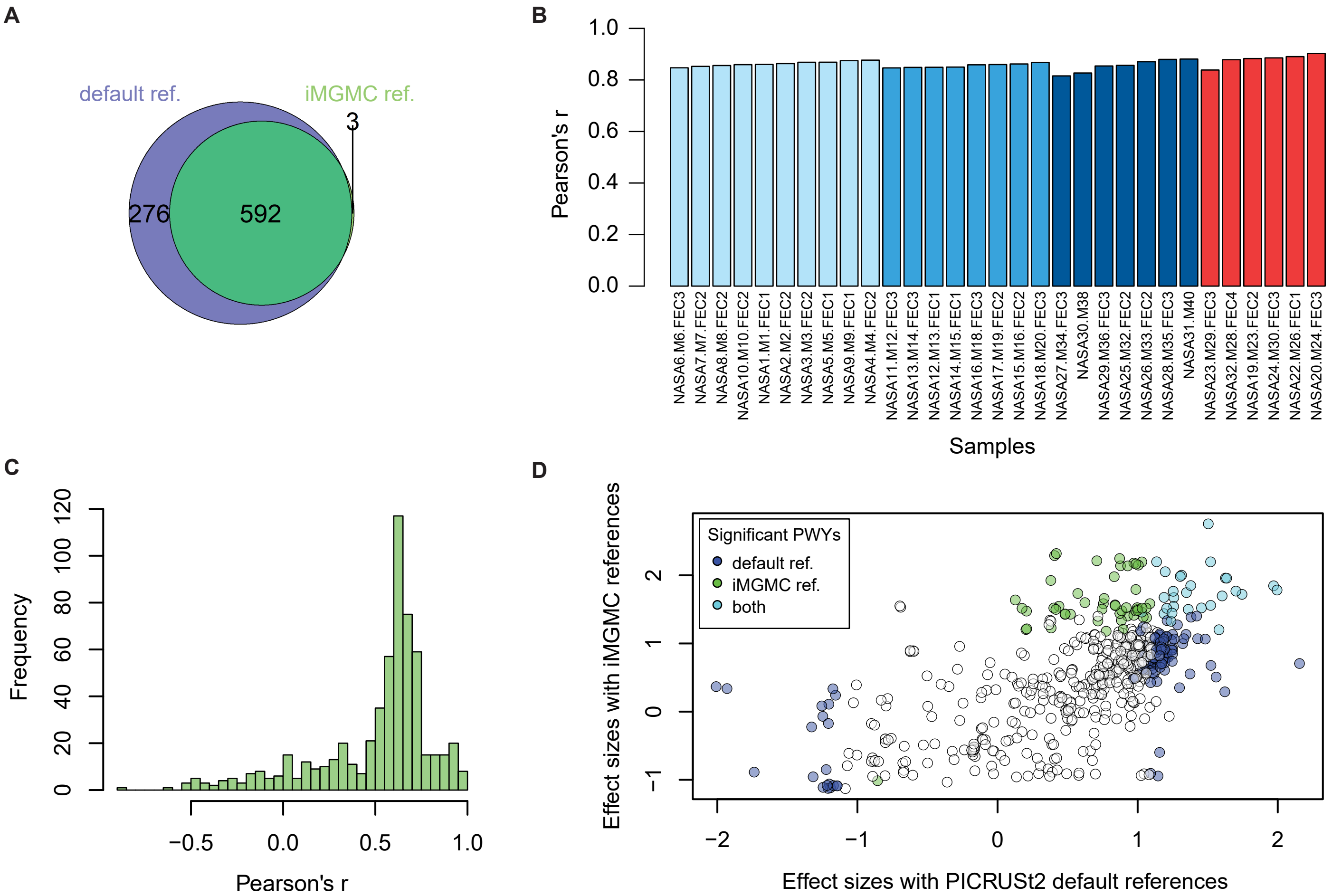

Figure S3. Comparison of inferred gene abundance of microbial pathways predicted using either the PICRUSt default reference or the iMGMC reference. (**A**) A Venn diagram showing the number of pathways uncovered with each of the reference catalogs. (**B**) Coefficients of pathway-wise correlations computed for each of the samples showing a high agreement in the predicted gene abundance of pathways uncovered by both reference catalogs. (**C**) Sample-wise correlations between the relative abundance predicted using the two reference catalogs were computed for each of the pathways, and a histogram is used here to show the distribution of correlation coefficients. CLR-transformed relative abundance was used to compute correlation coefficients. (**D**) A scatter plot showing the overall similarity (Pearson's r = 0.61, P = 6.98×10^{-61}) in the differential abundance effect sizes estimated by ALDEx2 using gene abundance predicted with either reference catalog. Each point denotes a pathway that commonly uncovered using both references, and points filled with colors indicate pathways found to be differentially abundant (FDR < 0.1, ALDEx2) when the abundance was predicted using either reference or both. The lower correlation coefficients in **C** and **D** compared to **B** was expected, as differences in the number of pathways uncovered with each reference influences the estimated total sequence abundance and thus the estimated relative abundance of each predicted pathway.
